# Modeling Costs and Life-Years Gained by Population-Wide Next-Generation Sequencing or Single-Gene Testing in Nonsquamous Non–Small-Cell Lung Cancer in the United States

**DOI:** 10.1200/PO.22.00294

**Published:** 2023-01-12

**Authors:** Christopher A. Lemmon, Jie Zhou, Brian Hobbs, Nathan A. Pennell

**Affiliations:** ^1^ Department of Hematology and Medical Oncology, Taussig Cancer Institute, Cleveland Clinic, Cleveland, OH; ^2^ Present address: University of Cincinnati College of Medicine, Department of Internal Medicine, Division of Hematology and Oncology, Cincinnati, OH; ^3^ Cleveland Clinic Lerner Research Institute, Department of Quantitative Health Sciences, Cleveland, OH; ^4^ Present address: Department of Neuroscience, Novartis, Cambridge, MA; ^5^ Present address: Department of Population Health, Dell Medical School, The University of Texas at Austin, Austin, TX

## Abstract

Many patients with actionable driver oncogenes (ADOs) are never identified and thus never receive targeted treatment. This study evaluated the economic impact and the potential life-years gained (LYG) that can be attributed to the extent of next-generation sequencing (NGS) testing in the United States compared with single-gene testing (SGT) in patients with metastatic nonsquamous non–small-cell lung cancer in the United States.

**METHODS:**

A model was developed to evaluate incremental rates of SGT or NSG testing on the basis of LYG and cost per LYG. ADOs included for NGS included *EGFR*, *ALK*, *ROS1*, *BRAF*, *RET*, *MET*, and *NTRK*. SGT included *EGFR* and *ALK*. Assumptions were made for expected incidence of ADOs. Survival distributions were fit to published trial averages of median and 5-year overall survival. Treatment costs were estimated from drug cost averages. Reimbursement costs were based on data from the Center for Medicare and Medicaid Services.

**RESULTS:**

Each incremental 10% increase in NGS testing produces an average of 2,627.4 additional LYG, with an average cost savings per LYG of $75 US dollars (USD). Replacing SGT at the current rate of 80% with NGS testing would result in an average additional 21,09.6 LYG and reduce cost per LYG by an average of $599 USD. If 100% of eligible patients were tested with NGS and each identified patient had matched treatment, the total average cost per LYG would be $16,641.57 USD.

**CONCLUSION:**

On the basis of current evidence, population-level simulations demonstrate that clinically relevant gains in survival with non-negligible reduction in costs are obtainable from widespread adoption of NGS testing and appropriate treatment matching for patients with advanced nonsquamous non–small-cell lung cancer.

## INTRODUCTION

Non–small-cell lung cancer (NSCLC) continues to be one of the most prevalent and most deadly cancers in the United States, despite recent improvements in treatment options.^1^ Of an estimated 236,740 newly diagnosed patients yearly, more than half will present with advanced stage disease at the time of diagnosis. Moreover, the majority of patients initially presenting with early-stage disease will experience recurrence or progression, necessitating treatment strategies commensurate with primary metastatic non–small-cell lung cancer.^2^

**CONTEXT**

**Key Objective**
Single-gene testing for just one or two genomic alterations in non–small-cell lung cancer (NSCLC) is still common despite guidelines recommending comprehensive testing. Inadequate testing has implications for the survivorship of patients with NSCLC who harbor actionable driver oncogenes. This study estimates the economic impact as well as expected life-years gained for varying levels of next-generation sequencing (NGS) testing.
**Knowledge Generated**
As NGS testing replaces single-gene testing, more patients with actionable driver oncogenes will be identified and matched to appropriate therapies. Widespread adoption of NGS testing yields clinically relevant gains in survival with non-negligible reductions in costs.
**Relevance**
Broad implementation of NGS testing for all guideline recommended actionable driver oncogenes in practices that currently perform limited genomic testing offers meaningful survival benefits to patients and is financially economical. NGS testing should be performed universally for all patients with newly diagnosed advanced nonsquamous NSCLC.

Nonsquamous NSCLC has become a disease increasingly classified by genomic alterations, with highly effective therapies targeted to several of altered actionable driver oncogenes (ADOs). Current US Food and Drug Administration–approved targeted agents now include *EGFR*, *ALK*, *ROS1*, *BRAF*, *MET*, *RET*, and a tumor agnostic designation for *NTRK* fusions.^3-12^ Additionally, *KRAS G12C* targeted therapy is now approved for use in the second-line setting, and antibody-drug conjugate therapy for human epidermal growth factor receptor 2 (*HER2*) mutation, *NRG1* fusion, and others may be on the horizon.^13-15^ Importantly, appropriately matched therapy has yielded benefits in treatment responses and survival, and has shown benefit over standard chemotherapy and immunotherapy treatments.^3,16,17^ Conversely, unidentified patients who harbor these alterations and proceed with traditional systemic treatment not only have worse responses and outcomes, but also have the potential for significant adverse events in the setting of immunotherapy followed by initiation of a targeted agent, highlighting the importance of upfront testing for these ADOs and matching with the appropriate therapy.^18-20^

Testing recommendations were initially introduced in 2013 to include *EGFR* and *ALK*.^21^ These have since expanded as new agents have been approved and now include guideline-recommended testing for *ROS1*, *BRAF*, *MET* exon 14 skipping mutations, and *RET*. Additional recommendations are made with respect to *KRAS*, *MET* amplification, *NTRK*, and tumor mutation burden.^22,23^

Despite this, uniform incorporation into clinical practice has been slow and inconsistent. Demonstrating this, *EGFR* testing rates in the early 2010s were < 20%, and this has improved to more than 80% in 2018.^24-27^ Similarly, *ALK* mutation testing has improved from 32% in 2011 to > 60% in 2018.^28^ However, complete recommendation guided testing still lags, with only 8% achieving this in 2016 and 18% in 2018. There is also wide variation between clinical settings in rates of testing and type of testing, with 59% of academic practices using multigene panels, while only 28% of community practices did.^29,30^

With more genomic variants being recommended for testing before treatment, next-generation sequencing (NGS) has been increasingly used, but single-gene testing (SGT) and sequential testing after limited panels or exclusionary testing remains common. A commonly referenced barrier to NGS testing is cost of testing and the potential need for treatment with expensive targeted agents.^28^

To better assess the utility of broad versus limited genomic testing in identifying ADO+ patients and measuring costs and benefits of treating them with targeted therapies versus missing the ADO and treating them only with standard therapy, we conducted statistical simulations to measure the population-level impact of various rates of NGS testing for patients with newly diagnosed advanced nonsquamous NSCLC in the United States where highly effective therapies are widely available to all patients.

## METHODS

**FIG 1.**
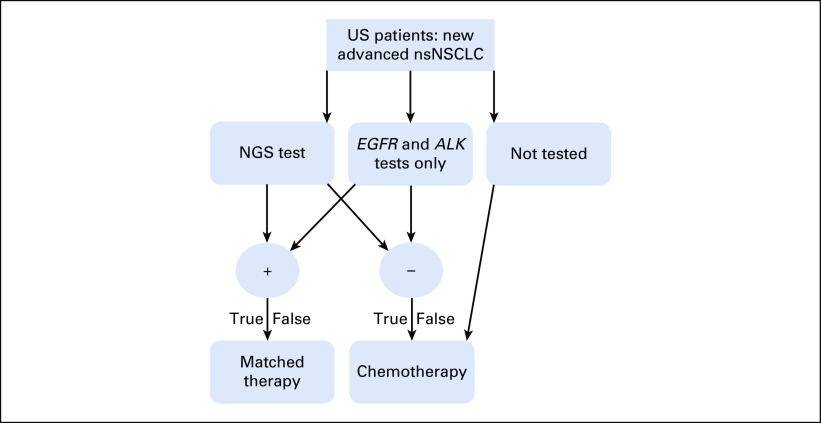
Diagram of the model structure. *ALK*, anaplastic lymphoma kinase; *EGFR*, epidermal growth factor receptor; NGS, next-generation sequencing; nsNSCLC, nonsquamous non–small-cell lung cancer.

A statistical model was constructed to simulate varying rates of testing with SGT or NGS with the objective of characterizing the population-level impact as measured by life-years gained (LYG) and cost per life-year gained (Fig 1). Estimated on the basis of the expected annual incidence rate in the United States at the time of study conception, we simulated a population of 89,000 hypothetical patients with newly diagnosed advanced nonsquamous NSCLC using our statistical model.^31^ The prevalence rate of actionable driver oncogenes in the patient population was estimated on the basis of current literature averages for the individual oncogenes.^27,29,30^ *EGFR* and *ALK* mutations were assayed as part of the SGT strategy on the basis of testing frequency trends established from literature, with an estimated 19% combined incidence rate.^28,29^ The NGS panel for the purpose of this study is defined as a panel including all ADOs recommended by the National Comprehensive Cancer Network at the time of study conception.^23^ These included *EGFR*, *ALK*, *ROS1*, *BRAF*, *RET*, *MET*, and *NTRK* with a combined estimated incidence rate of 29.3%. The overall testing rate is estimated to be 80% for *EGFR* and *ALK* mutations with approximately 15% of these because of NGS testing on the basis of current literature.^27,29^ We assume that 80% of patients with nonsquamous non–small-cell lung cancer in the United States undergo genetic testing of any type, while 20% are ineligible or do not receive testing.^29^ Among eligible patients, we simulate scenarios that reflect partitions of genetic testing rates from (0% NGS *v* 100% SGT) to (100% NGS *v* 0% SGT) in increments of 5%. Therefore, SGT and NGS rates vary from 0% to 80% of the total population. The sensitivity and specificity for each genetic test were assumed to be 100% for the purpose of this study. To elucidate results under the current state of the art, our figures delineate the scenario for which 15% of the total population receives NGS, while 65% receive SGT (dashed-red lines).

To determine appropriate assumptions for patient overall survival from the time of diagnosis and testing cost, we first carried out literature review of clinical trial survival outcome data for various targeted agents in patients with and without oncogene-driven advanced NSCLC, and reviewed cost estimates of the different drug treatments (Table 1). Although drug costs can be prohibitive to equitable access in some cases, this simulation study assumes that highly effective therapies are widely available to all patients.^33^ The Weibull distribution was used to determine survival duration for each simulated patient fit to statistical estimates of median and 5-year survival data from current literature.^3-12,16-18^ The fitted Weibull distribution yielded a median survival of 39 months with a 5-year survival of 25% for patients with an ADO that was identified and matched to targeted therapy. Patients harboring an unidentified ADO who received a non–ADO-based treatment strategy were estimated to have a median survival of 14 months and a 5-year survival of 5%. Only initial treatment was considered and no additional treatment lines were incorporated into the model because of the intrinsic heterogeneity of treatment patterns.

**TABLE 1.**
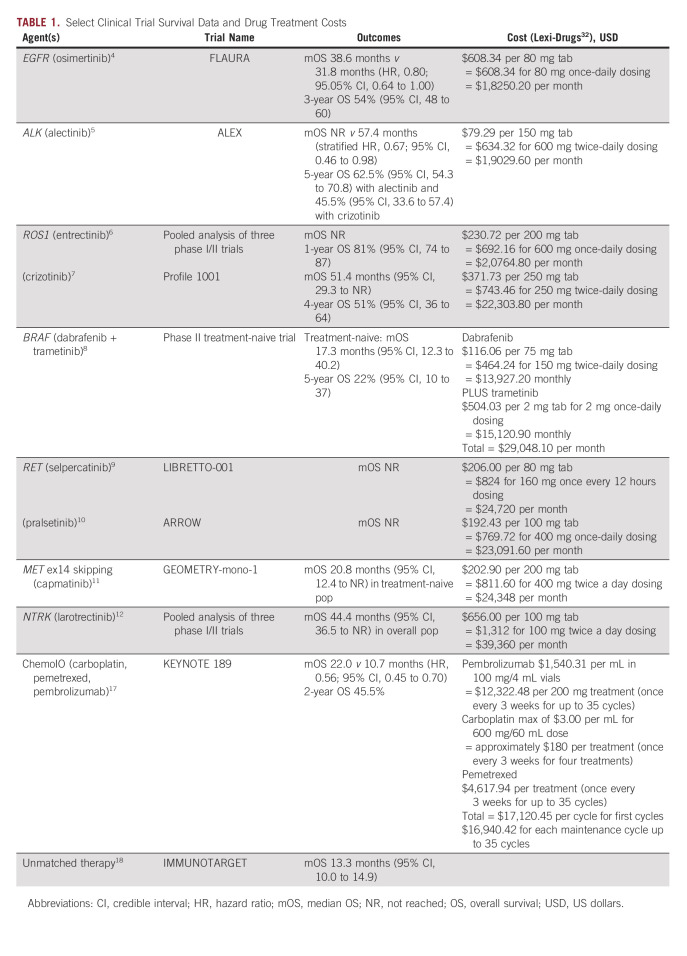
Select Clinical Trial Survival Data and Drug Treatment Costs

Cost estimates for different testing strategies were based on Center for Medicare and Medicaid Services (CMS) reimbursement figures, with SGT marked at $723.30 US dollars (USD) and NGS testing costing $627.50 USD.^34^ Drug treatment costs were based on estimated averages for both targeted therapies and chemoimmunotherapy as $10,000 USD per year (Table 2).^32^

**TABLE 2.**
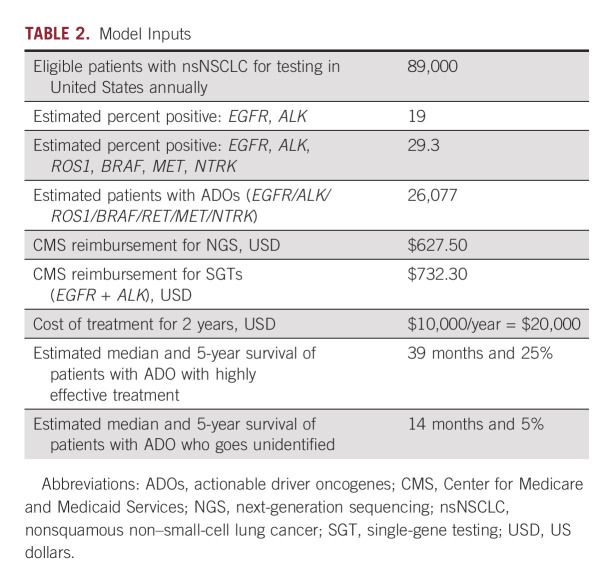
Model Inputs

The primary measure for this study was life-years gained with varying increments of NGS testing instead of SGT. A secondary measure was the cost per life-years gained with varying rates of NGS testing. The simulation study provides distributions for these measures. Estimates of life-years gained and cost results are reported as the distributional mean with 95% credible interval (CI) calculated from the resultant 2.5% and 97.5% percentiles.

## RESULTS

Our model generated simulations of 89,000 hypothetical patients with advanced nonsquamous NSCLC and configured testing strategies and costs to treatment strategy and outcome. In the absence of NGS testing (0% NGS) and assuming 80% of patients undergo SGT, a total of 15.2% of patients with nonsquamous NSCLC harboring an ADO were identified out of the total estimated 19% positive for *EGFR* or *ALK* mutations. Therefore, 3.8% of patients with *EGFR* or *ALK* mutations were unidentified because of overall lack of testing. All patients harboring non-*EGFR* or *ALK* ADOs remained unidentified, totaling 10.3% of the total estimated 29.3%. The 80% SGT strategy produced estimated total LYG of 38,838 (95% CI, 37,992 to 39,683), with an average total cost of $17,239 USD (95% CI, $17,067 to $17,408).

**FIG 2.**
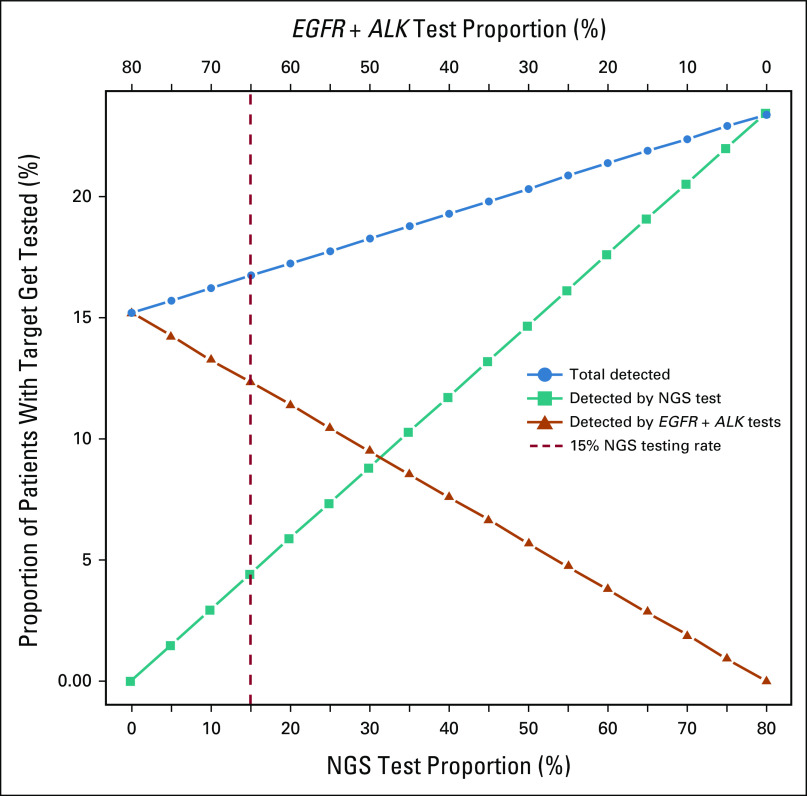
Assuming that 80% of patients with nonsquamous non–small-cell lung cancer are eligible for genetic testing, the rate at which patients with an actionable driver oncogene are identified increases from 15.2% when using SGT only to a maximum of 23.4% when NGS replaces SGT. The red dashed vertical line depicts the current state of the art, for which 15% of the total population receives NGS, while 65% receive SGT. *ALK*, anaplastic lymphoma kinase; EGFR, epidermal growth factor receptor; NGS, next-generation sequencing; SGT, single-gene testing.

As NGS testing replaced SGT, patients harboring an ADO were identified with increasing frequency. Fully replacing SGT with NGS among for eligible patients (80% NGS and 0% SGT) increased the number of ADO-positive patients identified from 15.2% to 23.4% (Fig 2). With this strategy, the average total LYG increases to 59,857 (95% CI, 58,905 to 60,808), with an average total cost of $16,639 USD (95% CI, $16,516 to $16,761). Therefore, at the current estimated rate of 80% testing for *EGFR* and *ALK* mutations, completely replacing SGT with NGS would result in an average of 21,019 additional LYG (95% CI, 19,746 to 22,291) and an average reduced cost per LYG of $599 USD (95% CI, $390 to $808).

**FIG 3.**
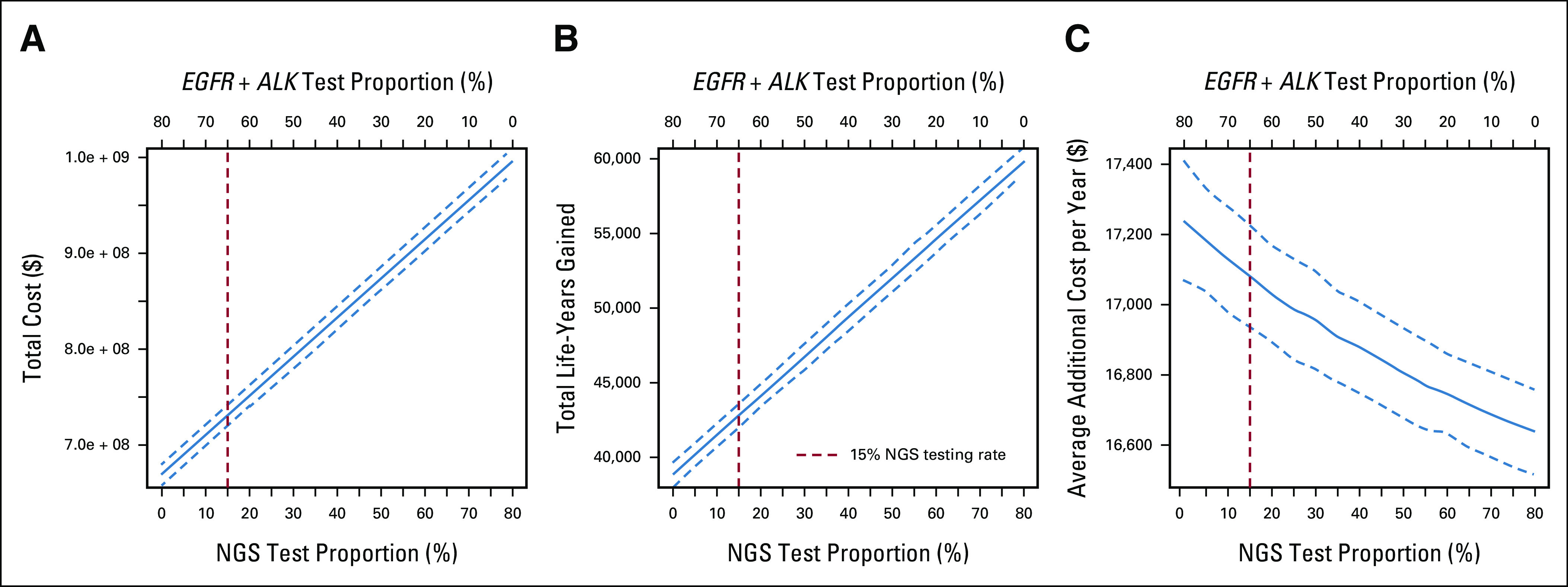
(A) Total costs, (B) life-years gained, and (C) average additional costs per year with 95% CIs for varying rates of SGT versus NGS assuming that a maximum of 80% of the nonsquamous non–small-cell lung cancer population is eligible for genetic testing. *ALK*, anaplastic lymphoma kinase; CI, credible interval; *EGFR*, epidermal growth factor receptor; NGS, next-generation sequencing.

As NGS-based testing supplanted SGT, each incremental 10% increase from 0% to 80% provided an average addition of 2,627.4 life-years gained with an average cost savings yield per life-year gained of $75 USD (Fig 3).

If NGS testing could be adopted for all patients, all 29.3% of patients with NSCLC harboring an ADO were able to be identified, adding an average of 15,017 LYG (95% CI, 13,729 to 16,304) when compared with testing 80% of the population. The total average cost per LYG under this scenario would be $16,642 USD (95% CI: $16,443 to $16,840).

## DISCUSSION

Highly effective targeted therapies are now available for an expanding number of actionable driver oncogene+ patients, but they can only be used and patients can only benefit if they are identified through testing. Because they often do not benefit from immunotherapy, the outlook for ADO-positive patients who go unidentified is similar to patients with advanced NSCLC treated with chemotherapy alone, making this identification exceedingly important. Simulation studies trained by current evidence were applied to interrogate the extent to which widespread adoptions of NGS testing have the potential to benefit patients with advanced nonsquamous NSCLC in the United States. This generated clinically meaningful gains in life-years that were attributable to incremental increases in NGS testing, and yielded a value-added benefit. The ability to provide matched targeted treatment to this patient population, conferring a significant survival benefit, amplifies the usefulness of broad testing with more economic benefit as patients live longer. As more driver oncogenes become druggable, these benefits would be expected to become more pronounced.

Previously, NGS testing was found to be more cost-effective than sequential and exclusionary testing, with the ability to save CMS $492,251 USD per year. A major contributor to the costs of SGT and exclusionary testing is having to test many genes sequentially. As the number of recommended genomic variants increased, costs associated with this repetitive testing increase, as well as the potential need for rebiopsy in the face of tissue utilization.^35^ The expansion of upfront NGS testing to replace SGT and sequential testing logically would eliminate these costs.

Another economic analysis comparing the total health care costs and value of treatment with appropriately matched targeted agents compared with standard therapy found that although there were increased costs of upfront treatment with the targeted agent, the improvement in survival yielded by ADO-based treatment eliminated this difference when costs were stratified per progression-free survival week.^36^

Additional benefits of broad NGS testing include the ability to better characterize the biology of the tumor to identify potential areas of study for clinical trials or potential future therapies, as well as provide potentially prognostic information.^37^ Noteworthy examples of this are the recent targetability of *KRAS G12C* mutations, and the intriguing use of antibody-drug conjugates for *HER2*-mutated NSCLC. These agents would affect the treatment on an additional 13% and 2%-4% of patients, respectively, and along with others, potentially provide further utility value in NGS testing and matched treatment.^3,13,14^

Continued efforts to analyze the value and benefits of different testing strategies should focus primarily on several areas. First, the characteristics of the NGS test used may play a significant role, as DNA-based NGS panels may miss a subset of driver oncogenes that could be identified with RNA-based testing.^38^ Using DNA- and RNA-based panels simultaneously or sequentially will need study to determine the efficacy and implications each strategy.

Second, the use of blood plasma circulating-tumor DNA panels using a liquid biopsy is well validated and now being increasingly used both in research and as standard practice in certain instances.^39^ Incorporation of the liquid biopsy may have broad implications, given the ability to expand access of NGS testing to those that do not have tissue available, preventing the need for rebiopsy.^40^

Finally, although molecular testing has mostly had treatment impacts in the metastatic setting, with the approval of osimertinib for adjuvant treatment in *EGFR*-mutant resected NSCLC, it is now important to expand this testing into early stages to capture these patients who harbor mutations to be able to provide matched therapy.^41^ Further study will be needed to determine the potential economic impact of SGT versus expanded NGS testing into earlier-stage lung cancers and the cost-effectiveness of ADO-targeted therapies in this setting.

Continued efforts to improve costs, turnaround time, testing sensitivity and infrastructure, and broad physician knowledge of guideline-based testing and treatment strategies are needed to provide additional economic and patient benefits.

This study is not without limitations. Simulation study requires appropriate assumptions and synthesis of evidence. Deviations from the underlying, unknown truth introduce source variability and bias. Our simulation was founded on the basis of a parametric statistical model for overall survival that was fit to statistical summaries of patient-level data reported in peer-reviewed clinical studies. The literature inherently reflects the expectations for patient survival on the basis of retrospective evidence. Additionally, the assumed costs for testing were delineated from CMS reimbursements, which does not take into account private payers. Moreover, the costs of treatment were generalized to a base average. The potential for extraneous variable costs that can be associated with patient care vary by clinical site and were excluded to enhance the generalizability of the simulation model. Decision branching for treatment was also limited to first-line management only, given the complex heterogeneity of treatment patterns at the time of progression.

In conclusion, because of the ability to identify more patients with actionable driver oncogenes and match them to the most appropriate initial therapy, clinically relevant gains in survival with appreciable reductions in costs can be achieved with widespread adoption of NGS testing for patients with advanced nsNSCLC. Our findings support a universal NGS testing strategy for all patients diagnosed with advanced nonsquamous NSCLC in the United States. Further interrogation is needed to determine the optimal methods for expanding NGS testing, integrating testing into earlier stages, and incorporating other modalities of broad testing such as RNA-based testing or circulating-tumor DNA-based liquid biopsy.
